# Thigh Ischemia-Reperfusion Model Does Not Accelerate Pulmonary VO_*2*_ Kinetics at High Intensity Cycling Exercise

**DOI:** 10.3389/fphys.2019.00160

**Published:** 2019-02-25

**Authors:** Lucas Helal, Paulo Cesar do Nascimento Salvador, Ricardo Dantas de Lucas, Luiz Guilherme Antonacci Guglielmo

**Affiliations:** ^1^Exercise Pathophysiology Laboratory, Hospital de Clinicas de Porto Alegre, Graduate Program in Cardiology and Cardiovascular Sciences, School of Medicine, Universidade Federal do Rio Grande do Sul, Porto Alegre, Brazil; ^2^Physical Effort Laboratory, Graduate Program in Biodynamics and Human Performance, Universidade Federal de Santa Catarina, Florianópolis, Brazil

**Keywords:** oxygen uptake kinetics, ischemia-reperfusion, cardiopulmonary test, physical exercise, exercise physiology

## Abstract

**Background:** We aimed to investigate the effect of a priming ischemia-reperfusion (IR) model on the kinetics of pulmonary oxygen uptake (VO_2_) and cardiopulmonary parameters after high-intensity exercise. Our primary outcome was the overall VO_2_ kinetics and secondary outcomes were heart rate (HR) and O_2_ pulse kinetics. We hypothesized that the IR model would accelerate VO_2_ and cardiopulmonary kinetics during the exercise.

**Methods:** 10 recreationally active men (25.7 ± 4.7 years; 79.3 ± 10.8 kg; 177 ± 5 cm; 44.5 ± 6.2 mL kg^−1^ min^−1^) performed a maximal incremental ramp test and four constant load sessions at the midpoint between ventilatory threshold and VO_2_ max on separate days: two without IR (CON) and two with IR (IR). The IR model consisted of a thigh bi-lateral occlusion for 15 min at a pressure of 250 mmHg, followed by 3 min off, before high-intensity exercise bouts.

**Results:** There were no significant differences for any VO_2_ kinetics parameters (VO_2_ base 1.08 ± 0.08 vs. 1.12 ± 0.06 L min^−1^; *P* = 0.30; τ = 50.1 ± 7.0 vs. 47.9 ± 6.4 s; *P* = 0.47), as well as for HR (MRT_180s_ 67.3 ± 6.0 vs. 71.3 ± 6.1 s; *P* = 0.54) and O_2_ pulse kinetics (MRT_180s_ 40.9 ± 3.9 vs. 48.2 ± 5.6 s; *P* = 0.31) between IR and CON conditions, respectively.

**Conclusion:** We concluded that the priming IR model used in this study had no influence on VO_2_, HR, and O_2_ pulse kinetics during high-intensity cycling exercise.

## Introduction

Pulmonary oxygen uptake (VO_2_) kinetics plays a role in the oxidative metabolism during constant load exercise (Poole et al., 2008). Considering that the area above the curve is accepted as a representative surrogate of the required additional energy supplying – i.e., anaerobic energy sources (Margaria et al., 1963), any acute or chronic adjustments that could reduce the time to attain a steady state of oxygen uptake and improves the energy imbalance becomes relevant for aerobic exercise performance (Gastin, 2001) or health related outcomes (Poole and Jones, 2012).

Several methods have been used to acutely challenge the VO_2_ control system and to enhance VO_2_ kinetics, such as breathing hyperoxic gas (MacDonald et al., 1998, 2000); prior exercise (Gerbino et al., 1996; Burnley et al., 2001, 2002) a reactive hyperemia induced by ischemia-reperfusion (IR) (Walsh et al., 2002; Faisal et al., 2010) – Which have resulted in controversial findings. The IR model involves blocking arterial to the target site (usually through a cuff pressure device), for a given period (continuously or intermittently). Upon cuff release, blood flow is augmented well above resting values (Kooijman et al., 2008). The brief exposure of muscle tissue to a blood flow absence causes an immediate reduction of the local O_2_ saturation, followed by a reduced adenosine triphosphate (ATP) and phosphocreatine (PCr) intramuscular concentrations, and an increase in adenosine diphosphate (ADP) and inorganic phosphate (Pi) intramuscular concentrations (Boushel et al., 1998). On the very end, an increase in vascular dilation (Enko et al., 2011), a potential increase in local blood flow, O_2_ muscle saturation and cardiac function (Daly and Bondurant, 1962; Faisal et al., 2010) is expected, which may accelerate VO_2_ kinetics by improving O_2_ delivery and availability. Nonetheless, the blood flow restriction (e.g., increases in ADP, Pi, etc.,) may affect the VO_2_ time course through spin-off manifestations, likely improvements in the mitochondrial oxidation activity through the increases in ADP and Pi concentrations (Boushel et al., 1998; Walsh et al., 2002). Despite the physiological rationale, the IR model has received little attention to study VO_2_ kinetics at the onset of exercise.

Few studies were conducted with contradictory and inconclusive findings, like an impairment (Faisal et al., 2010), no effect (Kido et al., 2015) or an improvement (Walsh et al., 2002) in the pulmonary VO_2_ kinetics. At high intensity exercise, VO_2_ kinetics appears to be sensitive to the fundamental component amplitude (Ap) (Walsh et al., 2002; Wilkerson et al., 2006), slow component amplitude (As) (MacDonald et al., 1998; Burnley et al., 2002; Walsh et al., 2002), time-constant of fundamental component (τ_p_) (MacDonald et al., 1998) and the “overall” kinetics (Walsh et al., 2002; Tordi et al., 2003; Nascimento et al., 2015). Also, VO_2_ kinetics is dependent on systemic and peripheral mechanisms, such as increased activity of oxidative enzymes, muscle blood flow and improved O_2_ distribution in the exercise muscles (Burnley et al., 2002; Wilkerson et al., 2006; Faisal et al., 2010; Poole and Jones, 2012). Notwithstanding, there is a question that still provokes further investigation for the physiologists: is the VO_2_ kinetics at the onset of exercise limited by muscle O_2_ delivery or by an oxidative enzyme inertia? In that scenario the reactive hyperemia by occluding target members, then augmenting muscle blood flow for several minutes prior to a subsequent exercise bout (Faisal et al., 2010), with less pronounced changes in the metabolic environment (i.e., peripheral mechanisms) seems an innovative approach.

Finally, it has been suggested that O_2_ delivery does not limit the phase II of VO_2_ kinetics in healthy subjects during a high intensity exercise (Poole et al., 2008), at least in some instances. However, at some “tipping point,” an O_2_ limitation could occur and VO_2_ kinetics can be modified through alterations in O_2_ availability. Specifically, high-intensity approaches the tipping point, where VO_2_ kinetics is O_2_-delivery dependent and may potentially be more susceptible to the influence of blood-muscle O_2_ influx (Poole and Jones, 2012). Thus, this study aims to investigate the effect of a priming IR protocol prior to high-intensity exercise on pulmonary VO_2_ kinetics and cardiac parameters (heart rate and O2 pulse). We hypothesized that a prior IR stimulus would accelerate VO2 and cardiac related kinetics at the onset of high-intensity cycling.

## Materials and Methods

### Participants

Ten recreationally active men (age 25 ± 5 years; body mass 79.3 ± 11 kg; height 177 ± 5 cm; VO_2_ max 44.5 ± 6.2 mL kg^−1^ min^−1^) volunteered to participate in this study. Participants were classified as physically active by the American College of Sports Medicine (ACSM) criteria (cardiorespiratory exercise training for ≥30 min⋅days^−1^ on ≥5 days⋅week^−1^ for a total of ≥150 min⋅week^−1^) (Garber et al., 2011). Some of them were involved in further recreational sport activities but not in any systematic physical exercise program. Participants were informed about risks and possible discomfort related to the protocol study. After that, a written informed consent was given before starting the study.

The subjects were instructed to avoid any intake of caffeine for 3 h, or alcohol and strenuous exercise in the 24 h preceding the test sessions. They were also instructed to arrive at the laboratory in a rested and fully hydrated state, and to eat the last meal up to 2 h prior to the experiment. All tests were conducted at the same time of day in controlled environmental laboratory conditions (19–22°C; 50–60% RH), to minimize the effects of diurnal biological variation on the results. All procedures were performed in accordance to the Declaration of Helsinki statement and were reviewed and approved by the Institutional Research Board (Ethics Committee for Research in Human Beings, Universidade Federal de Santa Catarina, IRB number 51045315.7.0000.0121).

### Experimental Design

The subjects visited the laboratory on five occasions. In the first visit, participants underwent a maximal incremental cycling test to determine intensity parameters for the experimental protocol (Visit 1); the other four experimental visits were randomly assigned, and separated by at least 48 h.

**FIGURE 1:**
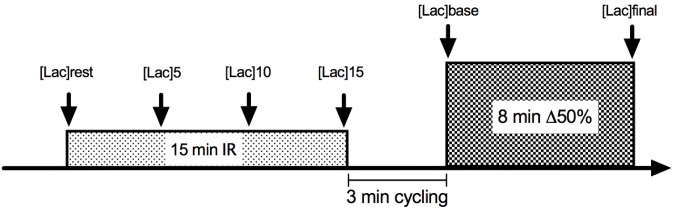
Illustrative figure of ischemia-reperfusion (IR) experimental design. For CON conditions, the experiment initiated with the 3-min 20 W cycling.

The experimental trials consisted of 3 min of baseline cycling at 20 W plus 8 min at Δ50% intensity. Gas exchange was collected throughout exercise bouts to analyze VO_2_, and O_2_ pulse kinetics. Heart rate (HR) was measured during the entire exercise bouts as well. In two of the four trials, cycling exercise was preceded by 15 min bilateral thigh blood flow occlusion, whereas two trials without IR were conducted as control sessions (Figure 1).

### VO_2_ max, GET and Δ50% Determination

To determine physiological indexes for exercise sessions, we conducted the maximal incremental ramp test on a cycle ergometer (Lode Excalibur, Groningen, Netherlands). Participants exercised for 4 min at a constant load of 20 W to stabilize gas measurements and then the exercise intensity increased systematically by 1 W every 2 s (rate of 30 W min^−1^) until volitional exhaustion.

Oxygen uptake was measured breath-by-breath using a gas analyzer (Quark PFT Ergo, COSMED, Rome, Italy). The gas analyzer was calibrated immediately before each test using ambient air and certificated alpha standard gasses containing 16.0% of oxygen and 5.0% of carbon dioxide. The turbine flowmeter was calibrated with a syringe of three liters (Quark PFT Ergo, COSMED, Rome, Italy). HR was continuously recorded by a HR monitor integrated into the gas analyzer system. Earlobe capillary blood lactate samples (25 μL) were taken before and immediately after the incremental exercise from the ear lobe and analyzed by an electrochemical method (YSI 2700 STAT, Yellow Springs, OH, United States).

Maximal oxygen uptake was defined by two criteria, depending on the presence of a VO_2_ plateau. The VO_2_ plateau was defined as any 60 s period in which the VO_2_ stabilized without increases higher than 150 mL min^−1^. In the absence of a VO_2_ plateau, we considered the maximum oxygen uptake as VO_2_ max, by the maximal observed value in a 15 s interval (Day et al., 2003). Maximal power output (Wpeak), maximal heart rate (HRmax) and maximal lactate were considered as the highest values observed during exercise. Gas exchange threshold (GET) was calculated using the V-slope method (Beaver et al., 1986). The increase in VO_2_ ventilatory equivalent (VE/VO_2_) with no further increases in VCO_2_ ventilatory equivalent (VE/VCO_2_) was verified through visual inspection and used as a secondary criterion.

The Δ50% exercise intensity was calculated by defining GET-VO_2_ and VO_2_ max, and then adding 50% of the difference between them to GET-VO_2_. After this, we made an interpolation using the linear regression model with the equation of a VO_2_/intensity relationship (Souza et al., 2016), in order to determine the Δ50% workload.

### VO_2_, HR, and O_2_ Pulse Kinetics

Each testing session consisted of a square-wave transition of 3 min at 20 W followed by 8 min at Δ50% intensity. Earlobe capillary blood lactate samples were collected 30 s before the baseline ([Lac]rest), 30 s before exercise ([Lac]pre) and immediately after the exercise ([Lac]post). O_2_ pulse was determined as the fraction between VO_2_ and HR (Whipp et al., 1996), both interpolated by 1 s intervals after matching the data of the two identical sessions corresponding to IR and CON conditions.

### Data Acquisition

Oxygen uptake and HR were recorded breath-by-breath during the entire exercise sessions (Quark PFT Ergo, COSMED, Rome, Italy) and were exported as raw data for filtering and analysis (OriginPro 7.0, United States). For VO_2_, signals suggesting sighs and coughs were initially removed from each test. According to Lamarra et al. (1987), occasional breath values were excluded from analysis using three standard deviations from the local mean as the criterion. Breath-by-breath data were linearly interpolated by 1 s intervals and then time-aligned to the start of exercise. After this procedure, both data series of each condition were matched and averaged in order to provide a unique profile for each exercise condition. The last filtering procedure was applied to reduce interpolated data to a 5 s stationary mean (Rossiter et al., 2005). The cardiopulmonary phase (i.e., first 20 s of exercise) was not included in the analysis (Rossiter et al., 2005).

For modeling the VO_2_ profile, a non-linear regression technique was applied (Rossiter et al., 2002), where phase II and a slow component were fitted separately. The phase II was modeled from the end of the cardio-dynamic phase to the beginning of the slow component phase. The slow component phase was determined by four criterias: (1) the narrowest confidence interval for τ; (2) breakpoint and the systematic rising of phase II amplitude and time-constant with a decrease in TD; (3) breakpoint and a systematic rising of fitting *R*^2^; (4) visual inspection of residual plot, considering the end-phase by the farthest point from zero. The model was constrained in the VO_2_ baseline (Barstow et al., 1996) to identify key parameters according to the Eq. 1:

Equation 1. Mono-exponential model for VO_2_ kinetics.

(1) VO2(t)=VO2 base+Ap[1−e−(t−TDp)τp]

where VO_2_ (t) represents VO_2_ values for a given time t; VO_2_ base is the average VO_2_ of the last minute of 20 W load period; Ap is the amplitude of phase II; τ_p_ is the phase II time-constant; and TD is the phase II time delay.

The slow component amplitude was determined by the difference between the VO_2_ end and the sum of the VO_2_ baseline and Ap, according to Eq. 2:

Equation 2. VO_2_ slow component calculation.

(2) As=VO2 end−(VO2 base+Ap)

Where, As is the slow component amplitude; VO_2_ end is the average VO_2_ value over the last 15 s; and Ap is the phase II amplitude.

The mean response time (MRT), which describes an overall pattern of VO_2_ kinetics for the exercise bout, was calculated by a mono-exponential model from the beginning to the end of the exercise, excluding the time delay (Barstow and Molé, 1991), according to Eq. 3. The same equation was used to fit HR and O_2_ pulse kinetics at the onset of the exercise (Wilkerson et al., 2006), with the end-point fixed at 180 s in order to augment fitting quality (i.e., better *r*^2^), since the bi-exponential pattern of HR and O_2_ pulse were not detected for all of the subjects:

Equation 3. Mean-response time (overall kinetics) for VO_2_, HR and O_2_ pulse.

(3) $${\mathrm{VO}}_{2}(t)={\mathrm{VO}}_{2}\mathrm{base}+\mathrm{Ap}\left[1-e\frac{-t}{\tau p}\right]$$

### Ischemia-Reperfusion Protocol

For IR, a 15 min bilateral thigh occlusion was introduced by the application of 250 mmHg pressure simultaneously in each thigh of the participants while they rested in a supine position (Ger-Ar, São Paulo, Brazil). Occlusion cuffs, chosen to be 20% wider than the upper leg diameter, were placed at the inguinal leg site. After 15 min of inflated cuffs, they were released, and the exercise load started following 3 min of 20 W cycling. Capillary blood lactate was taken from the ear lobe at rest and every 5 min until the end of the occlusion period ([Lac]_rest_, [Lac]_5_, [Lac]_10_, and [Lac]_15_). The occlusion time, mode and the time between cuff releasing and the beginning of exercise were determined based on a time-window where the blood flow remained augmented against the baseline conditions (Hampson and Piantadosi, 1988; Paganelli et al., 1989; Faisal et al., 2010). The period of time between the subject’s displacement from the bed to the cycle ergometer, as well as the data acquisition procedures (cuff displacement, face mask, HR strap adjustment, and sitting) were also controlled, in order to ensure trial reliability.

### Statistical Analysis

Descriptive data is presented as mean ± standard deviation. Firstly, we tested the normality of the distribution by the Shapiro-Wilk test (*n* < 50). For variance analysis, sphericity assumptions were tested by the Mauchly’s test, and corrections were made by the Greenhouse-Geisser factor. For VO_2_, HR, and O_2_ pulse kinetics parameter comparison, the two-tailed Student *t*-test for paired samples was used. Log transformations were made when necessary. For lactate concentration analysis between-conditions, the two-way ANOVA for repeated measures (condition vs. time) was conducted. For comparisons of lactate concentrations during thigh occlusion (in supine position), we used a one-way ANOVA for repeated measures. The Bonferroni’s *post hoc* analysis was used to estimate point-by-point differences after ANOVA. The sample size was calculated for a moderate effect size (*ES* = 0.5), with an α level of 0.05 and a statistical power of 80%, resulting in 10 subjects. VO_2_, HR and O_2_ pulse data were loaded into the Origin 8.0 software and the statistical analysis was completed using the SPSS 22.0 for Windows^®^ and the GraphPad Prism for Mac^®^ (Prism 7.0, United States). An α level of 0.05 was set to claim for statistical significance.

## Results

### Physiological Indexes

Table 1 shows the physiological indexes derived from the maximal incremental test.

**Table 1:** Physiological indexes obtained after maximal incremental test.

Variables	Mean ± SD	Minimum	Maximum
VO_2_ max (L min^−1^)	3.48 ° 0.40	2.70	4.06
Wmax (W)	320 ° 28	290	365
GET VO_2_ (L min^−1^)	2.03 ° 0.32	1.53	2.53
GET %VO_2_ max (%)	58.2 ° 5.20	48.20	63.60
GET W (W)	132 ° 24	110	170
GET %Wmax (%)	41.3 ° 5.40	37.60	53.10
Δ50% VO_2_ (L min^−1^)	2.75 ° 0.35	215	325
Δ50% %VO_2_ max (%)	79.1 ° 2.60	74.10	81.80
Δ50% W (W)	211 ° 28	163	249
Δ50% %Wmax (%)	65.8 ° 4.70	56.30	73

*VO_2_ max, maximal oxygen uptake; Wmax, maximal power output; GET, gas exchange threshold; Δ50%, GET plus 50% of the difference between it and VO_2_ max.*

### VO_2_ Kinetics at Δ50% After Ischemia-Reperfusion Model (IRT) and Control (CONT) Condition

**FIGURE 2:**
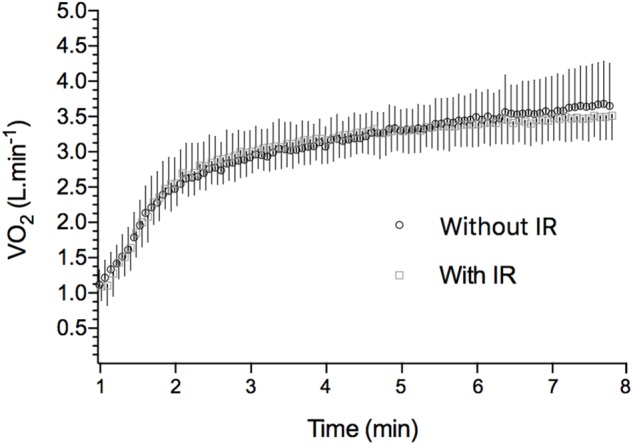
VO_2_ during high-intensity exercise in IR and CON conditions. Points are averaged in 5 s means along the exercise period (8 min). Each point is representative of the mean ± SD of all 10 studied subjects. Mean VO_2_ baseline for CON: 1.08 ± 0.08; mean VO_2_ baseline for IR: 1.12 ± 0.06.

**FIGURE 3:**
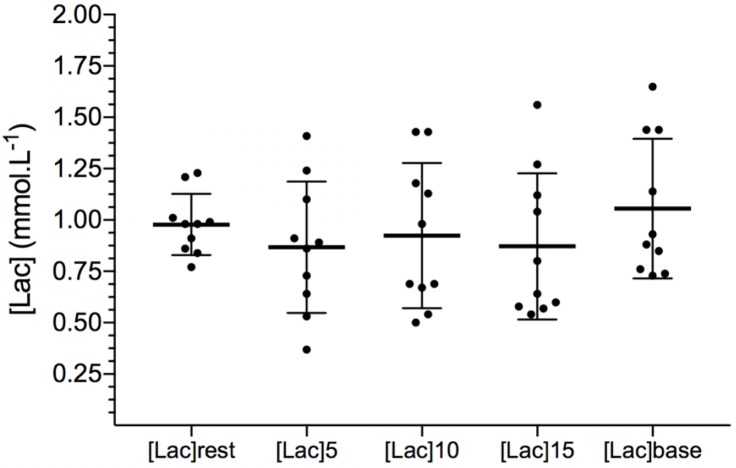
Capillary blood lactate concentrations during thigh blood flow restriction and after release. [Lac]base refers to the [Lac] immediately before the Δ50% exercise with individual observations. Data are expressed as mean ± SD.

Table 2 shows VO_2_ kinetics parameters obtained during the 8 min square wave exercise with or without previous limb IR protocol. In Figure 2, the overall exercise VO_2_ pattern is illustrated by averaging all the subject’s 5 s data points. VO_2_ measured at the end of square-wave trials were 3.38 ± 0.11 L min^−1^ and 3.41 ± 0.09 L min^−1^ (*P* = 0.39) for CON and IR conditions, respectively, overestimating predicted VO_2_ about 22.9% ± 0.3% and 24.1 ± 0.2%. There were no differences between the conditions for any VO_2_ kinetics parameters (VO_2_ base, A_p_, A_s_, VO_2_ end, τ_p_, TD_p_ e MRT). In addition, there were no interactional effects between the condition and time for the capillary blood lactate concentrations (*P* = 0.16), as well as no effect of conditions (CON vs. IR) (*P* = 0.20) for [Lac]rest (0.86 ± 0.32 and 1.16 ± 0.38 mmol L^−1^), [Lac]pre (1.04 ± 0.35 and 1.16 ± 0.44 mmol L^−1^) and [Lac]final (10.26 ± 1.62 and 9.43 ± 1.46 mmol L^−1^); and for [Lac] during bilateral thigh blood flow occlusion (*P* = 0.64; Figure 3).

**Table 2:** VO_2_ kinetics parameters.

Variables	CON	IR	*P*
Baseline			
VO_2_ base (L min^−1^)	1.08 ° 0.08	1.12 ° 0.06	0.30
Primary component			
A_p_ (L min^−1^)	2.07 ° 0.64	2.07 ° 0.48	0.99
τ_p_ (s)	50.1 ° 7.04	47.9 ° 6.37	0.47
TD_p_ (s)	6.11 ° 1.75	4.40 ° 1.2	0.34
Slow component			
A_s_ (L min^−1^)	0.22 ° 0.19	0.21 ° 0.17	0.75
Overall kinetics			
A_t_ (L min^−1^)	3.38 ° 0.11	3.41 ° 0.09	0.39
MRT_V O2_ (s)	76.6 ° 6.6	75.6 ° 5.2	0.75

*CON – exercise without previous IR; IR: exercise preceded by IR, VO_2_ base: average VO_2_ of the lasting 60 s of 20 W baseline load; A_p_, primary component amplitude; As, slow component amplitude; A_t_, overall amplitude; τ_p_, primary component time-constant; TD_p_, primary component time-delay; MRT, mean response time; α = 0.05.*

### HR and O_2_ Pulse Kinetics

Table 3 shows HR and O_2_ pulse kinetics results. MRT and the amplitude values are related to the time-fixed first 180 s of the exercise. There were no differences for any analyzed variables.

**Table 3:** HR and O_2_ kinetics.

Variables	CON	IR	*P*
HR			
HRbase (bpm)	93 ° 13	91 ° 11	0.57
A_180s_ (bpm)	61 ° 11	64 ° 9	0.10
MRT_180s_ (s)	67.25 ° 6.03	71.33 ° 6.08	0.54
O_2_ pulse			
(O_2_ pulse)base (mL bpm^−1^)	11.99 ° 0.81	12.29 ° 0.94	0.37
A_180s_ (mL bpm^−1^)	8.94 ° 0.48	9.96 ° 0.91	0.35
MRT_180s_ (s)	40.89 ° 3.85	48.15 ° 5.55	0.31

*HR base: heart rate of the lasting 60 s of the 20 W baseline load; (O_2_ pulse) base: O_2_ pulse of the lasting 60 s of the 20 W baseline load; A_180s_: fixed-time amplitude at 180 s after the onset of the exercise; MRT_180s_: time-constant of the variables fixed at 180 s after the onset of the exercise.*

## Discussion

### Main Findings

The main aim of this study was to investigate the impact of a prior bilateral thigh IR model on pulmonary VO_2_ kinetics during a cycling exercise at Δ50% intensity. The secondary aim was to investigate central control of the O2 pathway via the kinetic responses or HR and estimations of stroke volume (via O2 pulse) at the onset of exercise. Our major finding was that the proposed IR protocol did not modify VO_2_ kinetics during high-intensity exercise, neither HR and O_2_ pulse, rejecting our hypothesis that a previous blood flow occlusion protocol would accelerate pulmonary VO_2_ kinetics in a high intensity exercise. Our data also suggest that the IR model did not accelerate any central variables kinetics at the onset of the exercise. To the best of our knowledge, this is the first study to report the effects of IR on HR and O_2_ pulse kinetics during a high intensity cycling exercise.

In light of the previous studies in the literature related to IR model, the absence of effects is in accordance to the study of Kido et al. (2015), that did not find any acceleration of pulmonary VO_2_ kinetics during a severe intensity exercise when preceded by a similar protocol (ischemic preconditioning – 3 × 5 min bilateral occlusion, with 300 mmHg of pressure, ending within 5 min before exercise), especially at the onset of the exercise (τ_p_) and also for “overall” kinetics (MRT). Faisal et al. (2010) found a diminished muscle VO_2_ when the exercise began following 3 min of recovery after 15 min of forearm ischemia during an upper arm heavy exercise model, although an enhancement of forearm blood flow was observed. On the other hand, the findings of Walsh et al. (2002) suggests that “overall” pulmonary VO_2_ kinetics could be accelerated by previous thigh IR lasting 5 to 10 min using about 250 mmHg of pressure. However, the major difference between the Walsh et al. (2002) study to ours/others was the timing between the end of a blood flow occlusion and the beginning of exercise.

Whilst it has been suggested that the local muscular hyperemia could be accessed by up to 2–3 min after the cuff release (Mullen et al., 2001; Faisal et al., 2010), we consider this potential as a feature to explain possible differences. In our study, we chose a 3 min time frame until the beginning of the exercise based on a study of Faisal et al. (2010). In addition, the baseline exercise before the Δ50% exercise, which is recommended and often used for pulmonary VO_2_ kinetics experimental assessments (Rossiter et al., 2002; Poole and Jones, 2012), may have influenced muscle blood flow levels before Δ50% exercise as well.

A classic study of Daly and Bondurant (1962) provided accurate measurements of HR, cardiac output and stroke volume after 15 min of a 250 mmHg forearm blood flow occlusion, suggesting a sudden rise of those variables in the first 15 s of cuff releasing. Thus, the central parameter results, such as HR and stroke volume (O_2_ pulse), need to be considered when evaluating VO_2_ kinetics. Also, it is noteworthy to highlight that we did not find any effects of IR model on [Lac] during the occlusion period, neither any differences in [Lac] rest values and [Lac]pre. Blood lactate has been suggested as a properly mediator of O_2_ transport enhancement by vascular tissue dilation (Krustrup et al., 2001; Tordi et al., 2003), which could partially explain the VO_2_ kinetics acceleration after priming exercise conditions (Wilkerson et al., 2004; Nascimento et al., 2016). We also observed a concomitant absence of change in [Lac] and central hemodynamic parameters following the IT intervention. This may have occurred due to a higher than expected O_2_ delivery and availability during thigh occlusion, as well as a better mitochondrial oxidation caused by higher levels of ADP, Pi, and Cr, which would attenuate any changes in [Lac].

### Limitations and Future Directions

We considered that our findings need to be interpreted in light of its limitations. Here we point out some future directions for research that investigators could further consider.

When defining our protocol, we considered three major factors: (1) the pressure cuff level; (2) the time period for occlusion; (3) and the time between the end of occlusion and the beginning of the exercise. In regard to the first, a 250 mmHg level was chosen because of its efficiency to completely occlude the femoral artery blood flow (Sharma et al., 2014) and because it has been successfully by others (Salvador et al., 2015). For the same reason, the occlusion time was chosen – i.e., 15 min of bilateral thigh IR, as it had been shown to trigger reactive hyperemia (Kooijman et al., 2008) and necessary metabolic disturbances to maximize (Sharma et al., 2014). Finally, the time between the end of the IR model and the beginning of the exercise was selected for two main reasons: firstly, a physiological rationale to augment the local blood flow, supported by previous experimental results (Faisal et al., 2010); and secondly a logistic reason that is related to the necessary time to strictly adhere to the entire necessary methodological procedures for the VO_2_ kinetics assessments in cycle ergometry, such as the participants transposition from the supine to the upright position, moving to the ergometer, cuff deflation and equipment removal.

Our results reinforce the absence of the central limitation in the onset-exercise pulmonary VO_2_ kinetics, and, strengthen the evidence on this side of the discussion. Therefore, we recommend that further research limited to this aspect is unlikely to change the direction of the evidence by its robustness. However, investigators still interested in using IR to study the onset-exercise pulmonary or muscular VO_2_ kinetics, and should consider a reduction in the time to the beginning of the exercise and to control the confounding factors necessary to reduce muscle inertia and rest muscle tension (Hughson and Morrissey, 1983) such as the 3 min of a baseline exercise.

## Conclusion

We found that VO_2_, HR, and O_2_ pulse kinetics during a high-intensity cycling exercise was not accelerated when preceded by a 15 min bilateral thigh blood flow occlusion and 3 min off. These results have important implications in possible strategies to accelerate VO_2_ kinetics and improve metabolic efficiency. Considering there were no effects of IR when preceded by the off-period between the blood flow release and the exercise starting, further research should use other methods to investigate whether VO_2_ kinetics are limited by oxygen delivery or oxidative enzyme inertia.

## Ethics Statement

IRB: Comite de Ética em Pesquisa com Seres Humanos – Universidade Federal de Santa Catarina Procedures: we first submitted to IRB’s appreciation our project with rationale, methods, risks, and benefits. Together, the Consent Form was approved and, before any enrollment with this study, the subjects needed to assign it. In the consent form, information regarding risk, benefits, study’s rationale and relevance and their wrights to drop out of the study whenever they want was made available to them. All signed forms are still stored in the project’s folder.

## Author Contributions

All authors designed the study and interpreted the data. LH and PS collected and analyzed the data. LH wrote the first draft. PS, RdL, and LG wrote the final draft.

## Conflict of Interest Statement

The authors declare that the research was conducted in the absence of any commercial or financial relationships that could be construed as a potential conflict of interest.
